# Impact of physical activity on clinical outcomes in patients with liver cirrhosis: a prospective observational cohort study

**DOI:** 10.1038/s41598-026-55248-8

**Published:** 2026-06-08

**Authors:** Maha Elsabaawy, Mohamed Eissa, Mohamed Atef, Soad Rizk, Hend Awad, Basam M. Masoud, Amira M. Badawy, Madiha Naguib

**Affiliations:** 1 Department of Hepatology and Gastroenterology, National Liver Institute, Menoufia University, Shebeen El-Kom, Egypt; 2 Nutrition unit, National Liver Institute, Menoufia University, Shebeen Elkoom, Menoufia, Egypt; 3 Tropical Medicine Department, faculty of medicine, Menoufia university, Shebeen Elkoom, Menoufia, Egypt

**Keywords:** Cirrhosis, Physical activity, Sarcopenia, Prognosis, Survival, Risk stratification, Diseases, Gastroenterology, Medical research, Risk factors

## Abstract

Physical activity (PA) is a recognized determinant of outcomes in chronic diseases, yet its prognostic role in cirrhosis remains incompletely understood. To evaluate the relationship between habitual PA and liver disease severity, sarcopenia, and mortality in cirrhosis, and to develop a clinically applicable risk stratification model. In this prospective cohort study, 116 patients with cirrhosis were assessed for PA using the International PA Questionnaire–Short Form (IPAQ-SF), from which a PA Index (PAI; 0–7 scale) was derived. Clinical, laboratory, nutritional, imaging, and outcome data were obtained from routinely collected hospital electronic medical records and prospectively verified during scheduled follow-up visits. Predictors of 12-month mortality were identified using Cox proportional hazards models, and a risk-tiered classification system incorporating PAI was generated. Lower PAI was significantly associated with higher Child–Pugh scores (ρ = −0.28, *p* = 0.002), higher INR (*p* = 0.005), and lower albumin (*p* = 0.018). Sarcopenia was most prevalent among patients with low PAI (*p* < 0.001). Independent predictors of mortality included low PAI (HR = 2.12, 95% CI 1.22–3.68, *p* = 0.008), Child–Pugh class C, older age, hypoalbuminemia, sarcopenia, and sarcopenic obesity. The proposed risk-tiered model effectively discriminated against 1-year survival (low risk: 89.2%; moderate risk: 64.7%; high risk: 42.5%; very high risk: 18.2%; *p* < 0.001). The IPAQ-SF–derived PAI independently predicts mortality in cirrhosis and, when integrated with established prognostic factors, enhances risk stratification. Incorporating routine PA assessment into cirrhosis management may refine surveillance intensity, optimize transplant prioritization, and guide individualized prehabilitation strategies.

## Introduction

Liver cirrhosis represents the terminal stage of chronic liver disease and remains a major global health challenge, accounting for more than one million deaths annually worldwide^1^. Its clinical course is heterogeneous, ranging from compensated states to progressive decompensation with complications such as portal hypertension, ascites, hepatic encephalopathy, and sarcopenia—each of which markedly impairs survival and quality of life^2^. Accurate prognostication in cirrhosis is essential for risk stratification, treatment prioritization, and optimal allocation of liver transplantation resources.

Traditional prognostic models, including the Child–Pugh and Model for End-Stage Liver Disease (MELD) scores, predominantly rely on biochemical and laboratory parameters^3^. While these indices are valuable, they fail to capture extrahepatic determinants of outcome—such as physical performance and functional reserve—which have emerged as critical predictors of survival in chronic disease.

Physical activity (PA) and cardiorespiratory fitness are increasingly recognized as independent prognostic markers across a range of systemic illnesses, including cardiovascular, metabolic, and pulmonary disorders^4^. In patients with cirrhosis, physical inactivity is common, resulting from chronic inflammation, malnutrition, hormonal dysregulation, and muscle wasting. These factors contribute to frailty, sarcopenia, and adverse outcomes such as reduced quality of life, higher decompensation rates, and poor post-transplant recovery^5–8^. Despite this, the prognostic role of habitual physical activity and its integration into existing cirrhosis models remain underexplored.

The International Physical Activity Questionnaire–Short Form (IPAQ-SF) is a validated, reproducible, and easily administered instrument for estimating habitual activity levels^9^. Although extensively used in cardiovascular and metabolic studies, its predictive role in liver cirrhosis—particularly in relation to mortality, disease severity, and sarcopenia—has not been comprehensively examined.

Emerging evidence suggests that structured exercise in cirrhotic patients can improve muscle mass, reduce portal pressure, and enhance physical capacity^10^. However, optimal activity thresholds and their relationship with disease severity remain undefined.

Accordingly, this study aimed to examine the association between physical activity and major clinical outcomes in patients with liver cirrhosis—specifically mortality, disease severity, and sarcopenia prevalence—and to explore the potential utility of physical activity as an adjunct prognostic tool. By linking patient-reported habitual activity with objective biochemical and radiological parameters, we propose a functional framework that complements existing scoring systems and may improve prognostication, quality of life, and survival in this high-risk population.

## Methods

### Study design and population

This prospective observational study was conducted at the **National Liver Institute**,** Menoufia University**,** Egypt**, between **January 2022 and June 2023**. Consecutive adult patients with clinically or histologically confirmed liver cirrhosis were eligible for inclusion. The diagnosis was based on established clinical, biochemical, and imaging criteria.

Consecutive eligible patients attending hepatology outpatient clinics or admitted to inpatient hepatology services during the study period were screened for eligibility. Patients were identified through institutional clinical registries and hospital electronic records using documented diagnoses of liver cirrhosis based on ICD-compatible clinical diagnostic coding and physician-confirmed diagnosis.

This study was conducted in a cohort of patients with HCV-related cirrhosis, which constitutes the vast majority of cirrhosis cases in Egypt.

Exclusion criteria included:

1. Active hepatocellular carcinoma,
2. Severe hepatic encephalopathy precluding questionnaire completion,
3. Inability to provide informed consent.

Diagnostic definitions of cirrhosis, sarcopenia, and cirrhosis-related complications were based on internationally accepted clinical and radiological criteria. Physical activity assessment was performed using the validated IPAQ-SF instrument. CT-based skeletal muscle measurements were independently reviewed by experienced radiologists blinded to clinical outcomes whenever feasible.

All participants provided written informed consent prior to enrollment. The study protocol adhered to the **Declaration of Helsinki (2013 revision)** and received ethical approval from the **Institutional Review Board of the National Liver Institute**.

### Assessment of physical activity

PA was assessed using the **International Physical Activity Questionnaire–Short Form (IPAQ-SF)**, administered by trained interviewers to ensure comprehension and reduce reporting bias, particularly among patients at risk for minimal hepatic encephalopathy.

A **PAI** was calculated using reported intensity, frequency, and duration of activities, resulting in a 0–7 composite score. Based on IPAQ-SF categorical scoring guidelines^11^, patients were classified into:

- **Low activity**: No or insufficient activity to meet higher categories.
- **Moderate activity**: ≥3 days of vigorous activity ≥ 20 min/day or ≥ 5 days of moderate activity/walking ≥ 30 min/day.
- **High activity**: ≥3 days of vigorous activity or ≥ 7 days of combined walking/moderate/vigorous activity.

IPAQ-SF was selected for its validation, reliability across diverse populations, and feasibility in clinical settings for patients with chronic liver disease. IPAQ-SF assessments were administered by trained hepatology residents and research personnel using a standardized protocol. Interviewers were independent from the radiological sarcopenia assessment and were blinded to CT-derived skeletal muscle measurements at the time of questionnaire administration; however, complete blinding to routine clinical and laboratory parameters was not feasible within the clinical workflow.

Assessment of nutritional status: Nutritional status was evaluated using the Subjective Global Assessment (SGA) modified for liver disease (Royal Free Hospital SGA) as recommended by ESPEN guidelines for cirrhosis (12-13). Patients were classified as: • Well nourished (RFH-SGA-A) • Moderately malnourished or suspected of being malnourished (RFH-SGA-B) • Severely malnourished (RFH-SGA-C) These assessments were performed by trained dietitians from the Nutrition Unit at baseline, blinded to physical activity category whenever feasible.   Assessment of sarcopenia CT-derived skeletal muscle index measurements were independently performed by experienced radiologists using standardized L3 muscle area analysis. Radiologists were blinded to physical activity assessments and mortality outcomes during image evaluation.

The cross-sectional muscle area was normalized to height squared (cm²/m²). Sex-specific cutoff values were applied:

- < 50 cm²/m² for men.
- < 39 cm²/m² for women^12–14^.

**Sarcopenic obesity (SO)** was defined as the coexistence of sarcopenia with elevated body fat percentage (≥ 25% in men, ≥ 35% in women)^15,16^.

Body mass index (BMI) was calculated from measured weight and height using standard protocols.

### Assessment of liver disease severity

Disease severity was classified using the **Child–Pugh** and **MELD** scores^17,18^.

Additional recorded parameters included:

- **Clinical**: Presence of ascites, hepatic encephalopathy, variceal bleeding, spontaneous bacterial peritonitis, or hepatorenal syndrome.
- **Biochemical**: Bilirubin, albumin, ALT, AST, alkaline phosphatase.
- **Coagulation**: INR, prothrombin time (PT).
- **Renal**: Serum urea and creatinine.
- **Hematologic**: Hemoglobin, WBC, platelet count.

### Assessment of cirrhosis-related complications

Cirrhosis-related complications were systematically evaluated at baseline and during follow-up through clinical examination, laboratory investigations, imaging studies, and review of hospital records. Recorded complications included ascites, spontaneous bacterial peritonitis (SBP), hepatic encephalopathy (HE), hepatorenal syndrome (HRS), and gastroesophageal variceal bleeding.

Variceal bleeding was defined as clinically evident upper gastrointestinal hemorrhage confirmed by endoscopy demonstrating bleeding esophageal or gastric varices. Ascites was diagnosed by clinical examination and ultrasonography. Hepatic encephalopathy was diagnosed according to West Haven criteria. Spontaneous bacterial peritonitis was confirmed by ascitic fluid neutrophil count ≥ 250 cells/mm³ in the absence of secondary peritonitis. Hepatorenal syndrome was diagnosed according to contemporary International Club of Ascites criteria.

The frequency of cirrhosis-related complications was compared across physical activity categories to evaluate the relationship between habitual physical activity and clinical decompensation severity.

### Outcome measures

Participants were followed for **12 months** through clinic visits, telephone interviews, and review of hospital records.

- **Primary outcome**: 1-year all-cause mortality.
- Secondary outcomes included prevalence of sarcopenia and sarcopenic obesity, associations between physical activity and liver disease severity, and incidence/frequency of major cirrhosis-related complications including ascites, hepatic encephalopathy, spontaneous bacterial peritonitis, hepatorenal syndrome, and variceal bleeding.

### Data source and data collection

Data for this study were derived from routinely collected clinical records at the National Liver Institute, Menoufia University. Demographic, laboratory, radiological, and clinical outcome variables were extracted from hospital electronic medical records and standardized patient files by trained investigators using a predefined data collection sheet.

Physical activity assessment using the IPAQ-SF questionnaire was performed prospectively during outpatient clinic visits by trained interviewers.

CT-derived skeletal muscle measurements were obtained from clinically indicated abdominal imaging performed within three months of enrollment. CT-derived skeletal muscle index measurements were independently performed by experienced radiologists using standardized L3 muscle area analysis. Radiologists were blinded to physical activity assessments and mortality outcomes during image evaluation; however, complete blinding to all clinical data was not always feasible in the clinical setting.

Mortality and follow-up outcomes were verified through review of hospital records, scheduled clinic visits, and direct telephone communication with patients or their relatives.

### Data storage

Clinical, laboratory, radiological, and follow-up data were collected using standardized case report forms (CRFs) completed by trained investigators during patient enrollment and follow-up visits. Initial data collection was performed on paper-based CRFs and subsequently entered into a secure electronic database using Microsoft Excel spreadsheets accessible only to the research team. Data entry accuracy was verified by independent cross-checking against original medical records and imaging reports. All patient identifiers were removed before statistical analysis to ensure confidentiality and data protection in accordance with institutional ethical standards.

### Data management and quality control

Investigators had full access to the study data used in the analysis. Data extraction and entry were independently reviewed by two investigators to minimize transcription errors. Implausible or inconsistent values were verified through re-review of original medical records.

Data completeness was assessed before statistical analysis. Variables with missing or incomplete entries were cross-checked against hospital records whenever possible. Missing data were infrequent (< 5% for all analyzed variables). Complete-case analysis was therefore performed without imputation. No external database linkage was performed in this study.

Additionally, reliance on routinely collected clinical data may introduce misclassification bias and variability in documentation quality. Self-reported physical activity assessment may also be affected by recall bias despite interviewer-assisted administration.

### Sample size and power

The study sample was determined pragmatically from the eligible patient pool during the recruitment period. Post hoc power analysis indicated that with *N* = 116, the study achieved **> 80% power** to detect a **hazard ratio ≥ 2.0** for the effect of low vs. high PAI on mortality, at a two-sided α = 0.05^19,20^.

### Statistical analysis

**Fig. 1:**
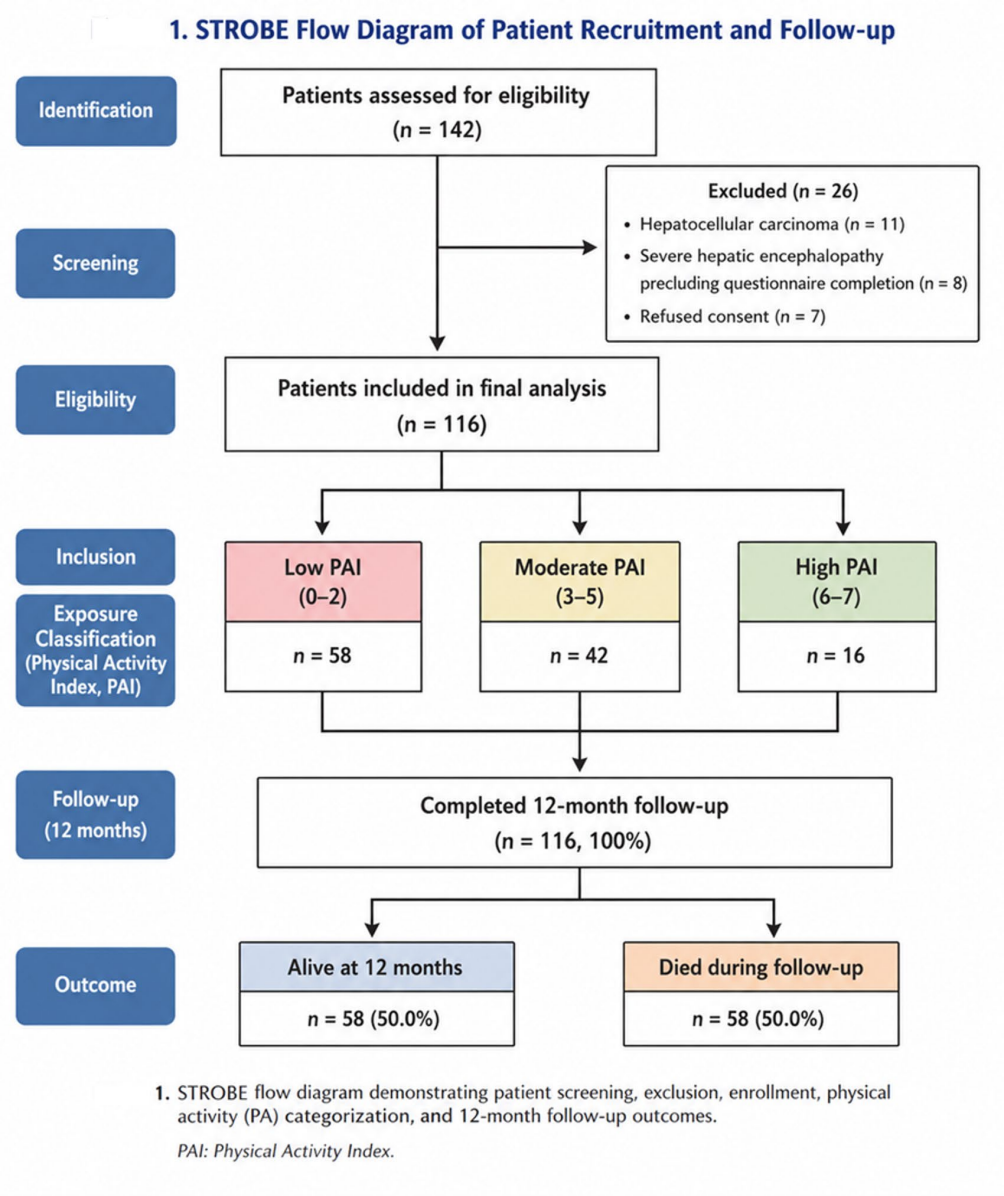
Flow chart diagram of patient recruitment and follow up. STROBE flow diagram demonstrates patient screening, exclusion, enrollment, physical activity categorization, and 12-month follow-up outcomes.

Statistical analyses were performed using IBM SPSS Statistics for Windows, Version 26.0 (IBM Corp., Armonk, NY, USA) and GraphPad Prism Version 9.0 (GraphPad Software, San Diego, CA, USA). Continuous variables are presented as mean ± standard deviation (SD) or median with interquartile range (IQR) according to data distribution, while categorical variables are expressed as frequencies and percentages. Group comparisons were performed using one-way ANOVA or Kruskal–Wallis tests for continuous variables and χ² test or Fisher’s exact test for categorical variables, as appropriate. Correlations between Physical Activity Index (PAI) and clinical variables were assessed using Spearman’s rank correlation coefficient. These analyses were exploratory and restricted to predefined clinically relevant parameters; therefore, formal correction for multiple comparisons was not routinely applied. Nonetheless, statistically significant findings were interpreted cautiously, particularly for associations with borderline p-values. Survival analysis was performed using Kaplan–Meier curves with log-rank testing. Multivariate Cox regression models were constructed using clinically relevant variables and those with univariate *p* < 0.10. Variables included age, Child–Pugh class, PAI category, albumin, sarcopenia, and sarcopenic obesity. Etiology was not included as all patients had HCV-related cirrhosis. Alcohol intake was negligible and therefore not modeled. Ascites and hepatic encephalopathy were not entered separately as they are core components of the Child–Pugh classification; however, sensitivity analyses forcing these variables into the model were performed. Figure 1.

## Results

### Baseline characteristics

This prospective cohort study included 116 patients with HCV-related liver cirrhosis. The mean age of the study population was 59.3 ± 11.2 years, and 68 patients (58.6%) were male. All participants had confirmed HCV-related cirrhosis. At baseline, the distribution of Child–Pugh classes was as follows: Class A in 18 patients (15.5%), Class B in 36 patients (31.0%), and Class C in 62 patients (53.4%). The mean MELD score for the entire cohort was 18.7 ± 6.5. Patients were categorized according to their Physical Activity Index (PAI) derived from the IPAQ-SF into three groups: low PAI (*n* = 58), moderate PAI (*n* = 42), and high PAI (*n* = 16). The mean body mass index (BMI) of the cohort was 27.6 ± 5.7 kg/m². Sarcopenia was present in 68 patients (58.6%), and sarcopenic obesity was identified in 31 patients (26.7%). Nutritional assessment by SGA revealed that 42 patients (36.2%) were well nourished (RFH-SGA-A), 48 (41.4%) moderately malnourished (RFH-SGA-B), and 26 (22.4%) severely malnourished (RFH-SGA-C).

Regarding decompensating features at baseline, ascites was absent in 41 patients (35.3%), mild-to-moderate in 52 patients (44.8%), and tense in 23 patients (19.8%). Hepatic encephalopathy (overt or minimal) was documented in 47 patients (40.5%).

Laboratory parameters at baseline for the whole cohort were as follows: mean serum albumin 2.8 ± 0.6 g/dL, median total bilirubin 1.9 mg/dL, and mean INR 1.5 ± 0.5 (Table 1).

Clinical and biochemical parameters differed significantly across the three Physical Activity Index (PAI) categories (low, moderate, and high activity groups). Overall, a progressive improvement in liver function and muscle status was observed with increasing physical activity levels. Patients with lower PAI demonstrated a higher prevalence of Child–Pugh class C (55% in low PA vs. 48% in moderate PA vs. 38% in high PA; *p* = 0.04), higher total bilirubin levels (2.1 ± 1.4 vs. 1.7 ± 1.1 vs. 1.3 ± 0.9 mg/dL; *p* = 0.03), lower albumin levels (2.7 ± 0.5 vs. 2.9 ± 0.5 vs. 3.1 ± 0.4 g/dL; *p* = 0.02), higher INR values (1.48 ± 0.38 vs. 1.35 ± 0.29 vs. 1.25 ± 0.22; *p* = 0.01), and lower skeletal muscle index (SMI) values (18.2 ± 3.9 vs. 19.5 ± 4.1 vs. 21.3 ± 3.5 kg/m²; *p* = 0.005) across low-, moderate-, and high-PA groups, respectively (Table 2).

### Correlation between PAI and disease severity

**Spearman correlation analysis** (Table 3) revealed significant negative associations between PAI and both **Child–Pugh score** (ρ = − 0.28, *p* = 0.002) and **total bilirubin** (ρ = − 0.25, *p* = 0.007), as well as between PAI and **INR** (ρ = − 0.26, *p* = 0.005). Conversely, PAI showed positive correlations with **serum albumin** (ρ = 0.22, *p* = 0.018) and **skeletal muscle index (SMI)** (ρ = 0.32, *p* < 0.001).

These findings suggest that greater habitual activity corresponds with preserved hepatic synthetic function and muscle mass.

### Follow-up outcomes and incidence of clinical events

During the 12-month follow-up period, all 116 enrolled patients completed outcome assessment through clinic visits, hospital record review, and telephone follow-up. Overall mortality during follow-up was 50.0% (58/116 patients).

Cirrhosis-related complications observed during follow-up included worsening ascites, hepatic encephalopathy, spontaneous bacterial peritonitis, hepatorenal syndrome, and gastroesophageal variceal bleeding. Ascites represented the most frequent complication, followed by hepatic encephalopathy and variceal bleeding (Table 4).

### Primary outcome: mortality

**Fig. 2:**
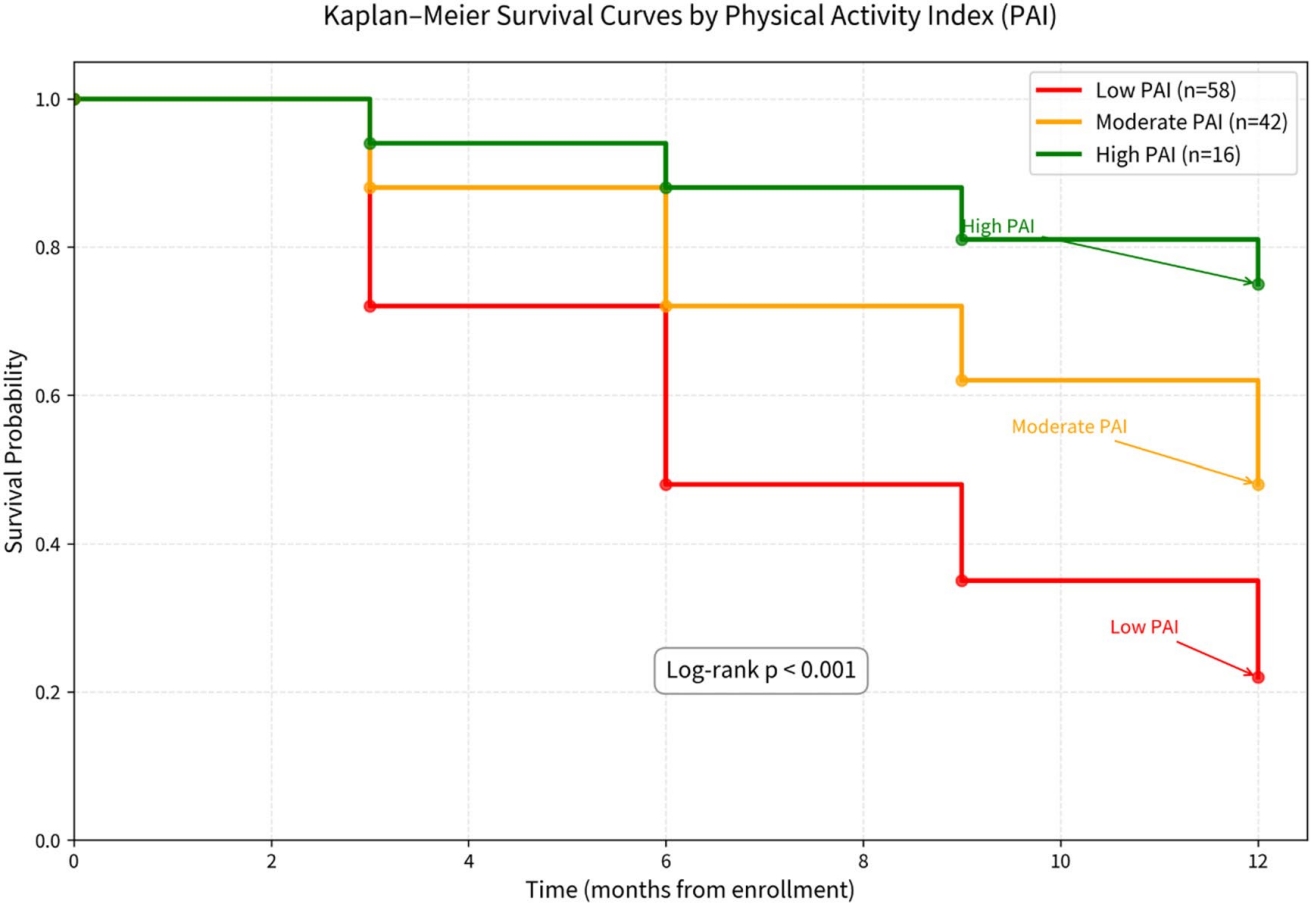
Kaplan–Meier survival curves stratified by Physical Activity Index (PAI) categories. Low PAI (red), Moderate PAI (orange), and High PAI (green). The curves demonstrate a clear dose-response relationship between physical activity level and survival over 12 months (log-rank test *p* < 0.001).

**Primary Outcome: Mortality** During the 12-month follow-up, 58 patients (50%) died. Patients who died had significantly lower median PAI [3 (IQR 1–4)] compared to survivors [5 (IQR 3–7); *p* < 0.001]. **Kaplan–Meier survival analysis** demonstrated a clear stepwise improvement in survival probability with increasing PAI categories (log-rank *p* < 0.001). Patients with high PAI had the best survival, while those with low PAI showed the steepest decline in survival. Survival curves stratified by the proposed risk-tiered classification system also showed excellent discrimination (log-rank *p* < 0.001) (Fig. 2).

### Secondary outcomes

**Fig. 3:**
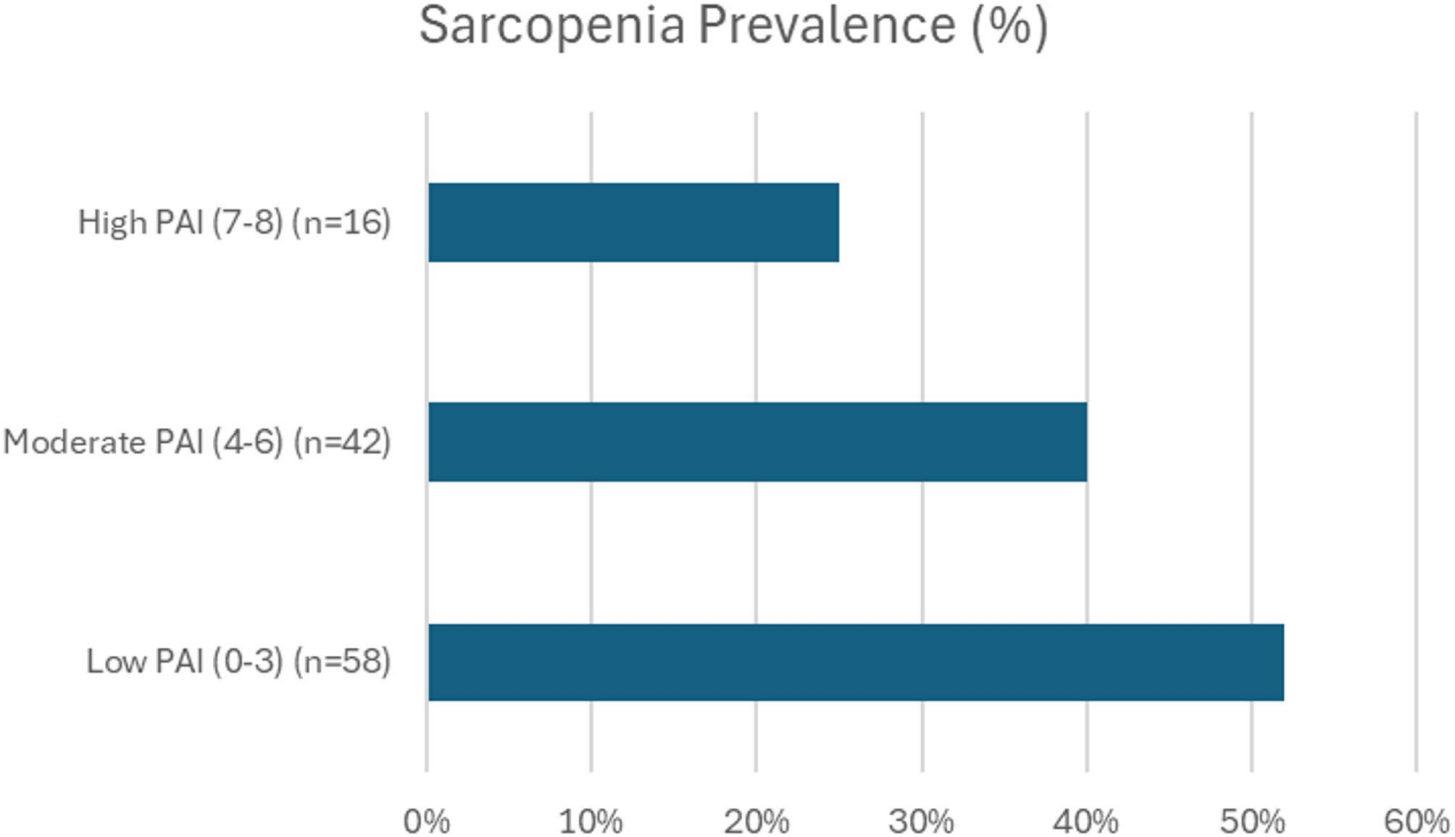
Prevalence of sarcopenia according to Physical Activity Index (PAI) groups. Sarcopenia was significantly more prevalent in the low PAI group (*p* < 0.001).

**Fig. 4:**
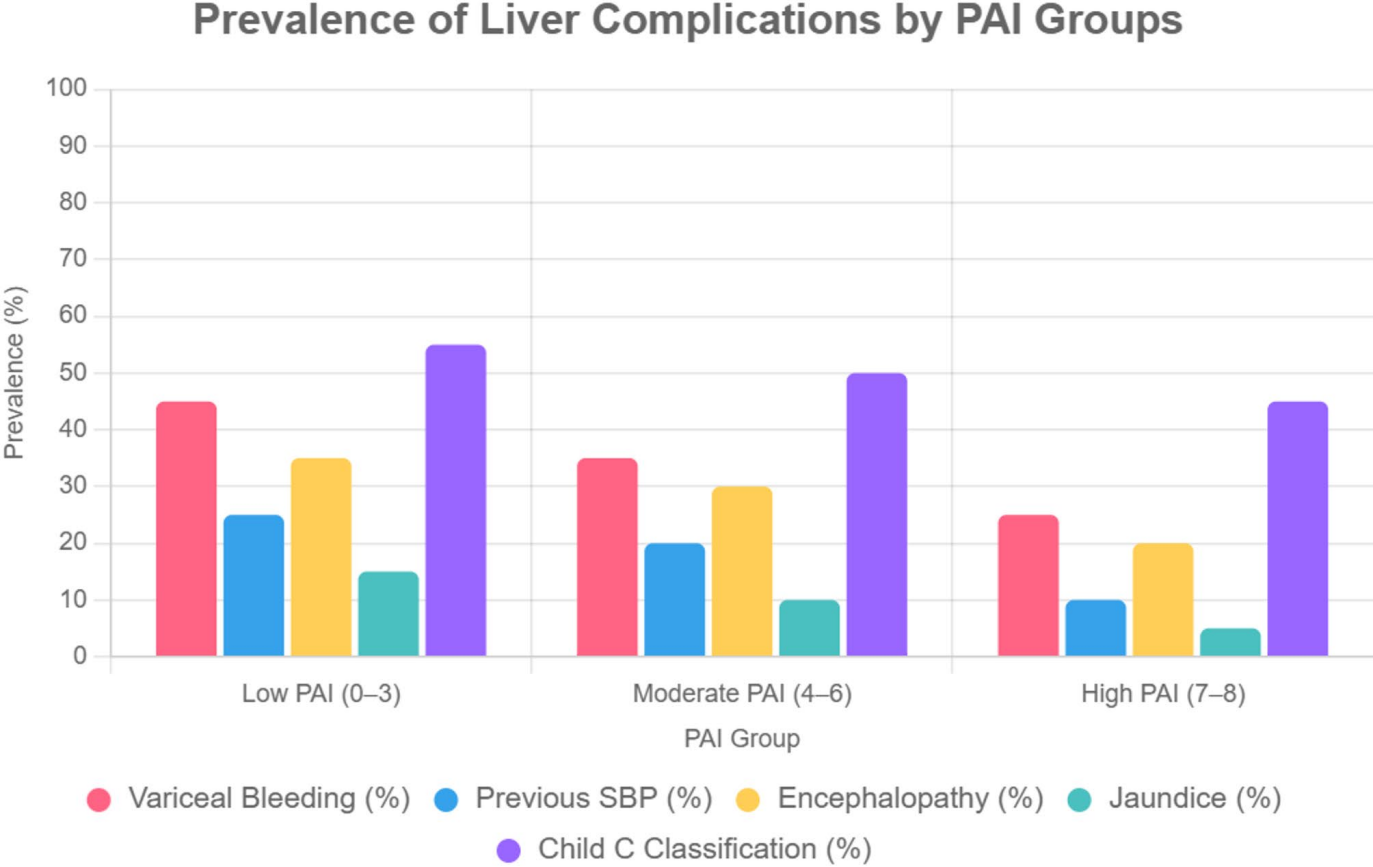
Prevalence of liver complications by PAI groups.

Sarcopenia was inversely related to PAI level, with prevalence highest among low-PAI patients and lowest among those with high activity (*p* < 0.001; Fig. 3). Similarly, liver-related complications such as **ascites** and **hepatic encephalopathy** were more frequent in patients with low PAI (Fig. 4). These patterns highlight the strong link between physical inactivity, muscle wasting, and clinical decompensation.

### Regression analysis

In **univariate Cox regression analysis** (Table 5), significant predictors of mortality included:

- **Age** (HR = 1.06; 95% CI 1.03–1.09; *p* < 0.001).
- **Child–Pugh class C** (HR = 3.45; 95% CI 2.01–5.92; *p* < 0.001).
- **Low PAI** (HR = 2.89; 95% CI 1.72–4.86; *p* < 0.001).
- **Lower albumin** (per 1 g/dL increase: HR = 0.42; 95% CI 0.29–0.61; *p* < 0.001).
- **Sarcopenia** (HR = 2.52; 95% CI 1.92–3.30; *p* < 0.001).
- **Sarcopenic obesity** (HR = 3.15; 95% CI 2.02–4.91; *p* < 0.001).• Nutritional status was strongly associated with mortality. Severe malnutrition (RFH-SGA-C) had HR 3.41 (95% CI 1.98–5.87, p

In **multivariate analysis**, independent predictors of 1-year mortality included (Table 6):

- **Age** (HR = 1.04; 95% CI 1.01–1.07; *p* = 0.012).
- **Child–Pugh class C** (HR = 2.78; 95% CI 1.58–4.89; *p* < 0.001).
- **Low PAI** (HR = 2.12; 95% CI 1.22–3.68; *p* = 0.008).
- **Albumin** (per 1 g/dL increase: HR = 0.58; 95% CI 0.39–0.87; *p* = 0.009).
- **Sarcopenia** (HR = 2.03; 95% CI 1.52–2.71; *p* < 0.001).
- **Sarcopenic obesity** (HR = 2.87; 95% CI 1.82–4.52; *p* < 0.001)• Nutritional status (RFH-SGA-C) remained independent in multivariate models (adjusted HR 2.31, 95% CI 1.28–4.17, p=0.005) (Table 6).

These findings confirm that lower physical activity independently predicts mortality even after controlling for hepatic functional status and nutritional parameters.

### Risk stratification

Integration of PAI with clinical and demographic parameters yielded a **risk-tiered classification system** that effectively discriminated against survival outcomes (Table 7).

- **Low-risk**: Child–Pugh A/B + PAI > 3 → 1-year survival **89.2%**.
- **Moderate-risk**: Child–Pugh B + Low PAI or Age ≥ 60 + High PAI → **64.7%**.
- **High-risk**: Child–Pugh C + High PAI + Sarcopenia → **42.5%**.
- **Very high-risk**: Child–Pugh C + Low PAI + Age ≥ 60 + Sarcopenia + RFH-SGA-C→ **18.2%** (*p* < 0.001).

**Table 1:** Baseline characteristics of the study cohort (*N* = 116).

Variable	Value
Age (years), mean ± SD	59.3 ± 11.2
Male sex, n (%)	68 (58.6%)
Etiology (HCV-related), n (%)	116 (100%)
Child–Pugh Class	
Class A, n (%)	18 (15.5%)
Class B, n (%)	36 (31.0%)
Class C, n (%)	62 (53.4%)
MELD score, mean ± SD	18.7 ± 6.5
BMI (kg/m²), mean ± SD	27.6 ± 5.7
Sarcopenia, n (%)	68 (58.6%)
Sarcopenic obesity, n (%)	31 (26.7%)
Ascites	
None, n (%)	41 (35.3%)
Mild-to-moderate, n (%)	52 (44.8%)
Tense, n (%)	23 (19.8%)
Hepatic encephalopathy, n (%)	47 (40.5%)
Albumin (g/dL), mean ± SD	2.8 ± 0.6
Total bilirubin (mg/dL), median	1.9
INR, mean ± SD	1.5 ± 0.5
PAI categories	
Low PAI, n (%)	58 (50.0%)
Moderate PAI, n (%)	42 (36.2%)
High PAI, n (%)	16 (13.8%)

SD = standard deviation; n = number; HCV = hepatitis C virus; MELD = Model for End-Stage Liver Disease; BMI = body mass index; INR = international normalized ratio; PAI = physical activity index.

**Table 2:** Baseline characteristics by physical activity groups.

Characteristic	Low PA (*n* = 58)	Moderate PA (*n* = 42)	High PA (*n* = 16)	*p*-value
Age (mean ± SD, years)	60.2 ± 11.8	57.9 ± 11.4	55.1 ± 10.2	0.12
Sex (Male, %)	53.4%	59.5%	68.8%	0.28
BMI (mean ± SD, kg/m²)	28.4 ± 6.1	27.1 ± 5.3	25.6 ± 4.7	0.09
Child–Pugh Class A (%)	10.3%	16.7%	31.3%	0.04
Child–Pugh Class B (%)	34.5%	35.7%	31.3%	-
Child–Pugh Class C (%)	55.2%	47.6%	37.5%	0.04
MELD score (mean ± SD)	20.4 ± 6.8	18.2 ± 6.1	15.9 ± 5.7	0.012
Etiology (HCV-related, %)	100%	100%	100%	-
Sarcopenia (%)	79.3%	52.4%	18.8%	< 0.001
Sarcopenic obesity (%)	36.2%	23.8%	6.3%	0.02
Ascites (None/Mild-moderate/Tense) %	22.4/48.3/29.3	38.1/45.2/16.7	68.8/25.0/6.3	0.008
Hepatic encephalopathy (%)	53.4%	35.7%	12.5%	0.015
INR (mean ± SD)	1.48 ± 0.38	1.35 ± 0.29	1.25 ± 0.22	0.01
Albumin (mean ± SD, g/dL)	2.7 ± 0.5	2.9 ± 0.5	3.1 ± 0.4	0.02
Bilirubin (mean ± SD, mg/dL)	2.1 ± 1.4	1.7 ± 1.1	1.3 ± 0.9	0.03
**SMI (mean ± SD**,** cm²/m²)**	**35.3 ± 4.1**	**39.8 ± 4.5**	**44.5 ± 3.8**	**< 0.001**

PA = physical activity; SD = standard deviation; BMI = body mass index; MELD = Model for End-Stage Liver Disease; HCV = hepatitis C virus; INR = international normalized ratio; SMI = skeletal muscle index.

**Table 3:** Correlation between PA and disease severity markers.

Marker	Spearman’s ρ	*p*-value
Child-Pugh Score (B = 0, C = 1)	−0.28	0.002
Bilirubin Total	−0.25	0.007
Albumin	0.22	0.018
INR	−0.26	0.005
SMI	0.32	< 0.001

PA, physical activity; INR, international normalized ratio; SMI; skeletal muscle index.

**Table 4:** Incidence of major complications during 12-month follow-up according to physical activity group.

Complication	Total (*n* = 116)	Low PA (*n* = 58)	Moderate PA (*n* = 42)	High PA (*n* = 16)	*p*-value
New or worsening ascites	38 (32.8%)	27 (46.6%)	9 (21.4%)	2 (12.5%)	0.003
New or worsening hepatic encephalopathy	29 (25.0%)	21 (36.2%)	7 (16.7%)	1 (6.3%)	0.008
Variceal bleeding	14 (12.1%)	10 (17.2%)	3 (7.1%)	1 (6.3%)	0.19
Spontaneous bacterial peritonitis	11 (9.5%)	9 (15.5%)	2 (4.8%)	0 (0%)	0.04
Hepatorenal syndrome	9 (7.8%)	7 (12.1%)	2 (4.8%)	0 (0%)	0.12
**Any major complication**	**72 (62.1%)**	**44 (75.9%)**	**22 (52.4%)**	**6 (37.5%)**	**< 0.001**

PA = physical activity; n = number.

**Table 5:** Mortality relations with different parameters.

Variable	Total (*n* = 116)	Alive (*n* = 58)	Dead (*n* = 58)	*p*-value
Demographics				
Age (years), mean ± SD	59.3 ± 11.2	55.1 ± 10.8	63.5 ± 10.1	< 0.001
Male sex, n (%)	68 (58.6%)	38 (65.5%)	30 (51.7%)	0.142
**Physical activity**				
PAI, median (IQR)	4 (2–6)	5 (3–7)	3 (1–4)	< 0.001
**Liver disease severity**				
Child–Pugh Class A, n (%)	18 (15.5%)	15 (25.9%)	3 (5.2%)	< 0.001
Child–Pugh Class B, n (%)	36 (31.0%)	25 (43.1%)	11 (19.0%)	< 0.001
Child–Pugh Class C, n (%)	62 (53.4%)	18 (31.0%)	44 (75.9%)	< 0.001
MELD score, mean ± SD	18.7 ± 6.5	15.3 ± 4.9	22.1 ± 6.1	< 0.001
**Etiology**				
Hepatitis C virus, n (%)	116 (100%)	58 (100%)	58 (100%)	-
**Nutritional status**				
BMI (kg/m²), mean ± SD	27.6 ± 5.7	26.8 ± 5.4	28.4 ± 6.0	0.14
Sarcopenia, n (%)	68 (58.6%)	22 (37.9%)	46 (79.3%)	< 0.001
Sarcopenic obesity, n (%)	31 (26.7%)	8 (13.8%)	23 (39.7%)	0.002
**Complications**				
Ascites – None, n (%)	41 (35.3%)	29 (50.0%)	12 (20.7%)	< 0.001
Ascites – Mild/Moderate, n (%)	52 (44.8%)	22 (37.9%)	30 (51.7%)	< 0.001
Ascites – Tense, n (%)	23 (19.8%)	7 (12.1%)	16 (27.6%)	< 0.001
Hepatic encephalopathy, n (%)	47 (40.5%)	14 (24.1%)	33 (56.9%)	< 0.001
**Laboratory values**				
Albumin (g/dL), mean ± SD	2.8 ± 0.6	3.0 ± 0.5	2.5 ± 0.6	< 0.001
Bilirubin (mg/dL), median	1.9	1.2	3.1	< 0.001
INR, mean ± SD	1.5 ± 0.5	1.3 ± 0.3	1.7 ± 0.6	< 0.001

PAI, physical activity index; MELD, Model for End-Stage Liver Disease; INR, international normalized ratio; SD, standard deviation; IQR, interquartile range.

**Table 6:** Mortality predictors (cox regression).

Predictor	Unadjusted HR (95% CI)	*p*-value	Adjusted HR (95% CI)	*p*-value
Age (per year)	1.06 (1.03–1.09)	< 0.001*	1.04 (1.01–1.07)	0.012*
Child-Pugh C	3.45 (2.01–5.92)	< 0.001*	2.78 (1.58–4.89)	< 0.001*
Low PAI	2.89 (1.72–4.86)	< 0.001*	2.12 (1.22–3.68)	0.008*
Albumin (per 1 g/dL ↑)	0.42 (0.29–0.61)	< 0.001*	0.58 (0.39–0.87)	0.009*
Sarcopenia (present)	2.52 (1.92–3.30)	< 0.001*	2.03 (1.52–2.71)	< 0.001*
Sarcopenic obesity	3.15 (2.02–4.91)	< 0.001*	2.87 (1.82–4.52)	< 0.001*

HR, hazard ratio; CI, confidence interval; PAI, physical activity index.

**Table 7:** Risk-tiered classification system.

Risk tier	Criteria	Number of patients	1-year survival	Suggested clinical actions
Low Risk	Child-Pugh A/B + High PAI > 3	34	89.2%	Routine monitoring q6mo
Moderate Risk	Child-Pugh B + Low PAI OR Age ≥ 60 + High PAI	29	64.7%	Enhanced monitoring q3mo
High Risk	Child-Pugh C + High PAI + Sarcopenia	28	42.5%	Prioritize for transplant evaluation
Very High Risk	Child-Pugh C + Low PAI + Age ≥ 60 + Sarcopenia	25	18.2%	Palliative care referral

PAI, physical activity index; q3mo, every 3 months; q6mo, every 6 months.

## Discussion

In this prospective cohort study of patients with liver cirrhosis, **lower PA** emerged as a robust and independent determinant of adverse outcomes, including greater disease severity, higher prevalence of sarcopenia, and increased mortality. Importantly, these associations persisted after adjustment for traditional prognostic factors such as age, Child–Pugh class, albumin, and sarcopenia, emphasizing that habitual physical activity represents an **independent physiological dimension of cirrhosis prognosis**.

Conventional models like the **Child–Pugh** and **MELD** scores remain foundational tools for assessing hepatic function; however, they largely overlook functional and systemic aspects of patient status^3^. Our results indicate that integrating a functional metric—such as the **IPAQ-SF–derived Physical Activity Index (PAI)**—provides incremental prognostic value. This reflects the growing recognition that extrahepatic fitness and physical reserve are critical to survival, paralleling findings in cardiovascular and metabolic disorders^4^.

The **inverse correlations** between PAI and established severity indices (Child–Pugh, bilirubin, INR) suggest that physical activity mirrors not only functional capacity but also underlying metabolic resilience. These results align with prior studies showing that diminished physical activity correlates with worse hepatic function and frailty, even after biochemical compensation^21–23^.

The strong relationship between low PAI and **sarcopenia** underscores the biological link between inactivity and muscle catabolism in cirrhosis. Inflammatory cytokines, hyperammonemia, and anabolic resistance contribute to progressive muscle loss, while inactivity exacerbates this cycle^24^. Our data reinforced earlier findings by **Montano-Loza et al.**^22^ and **Bhanji et al.**^17^, who demonstrated that sarcopenia independently predicts mortality even when MELD is accounted for.

Moreover, the observed association between low PAI and **sarcopenic obesity** highlights a high-risk phenotype marked by reduced muscle mass yet increased fat deposition—an increasingly recognized marker of metabolic frailty^15,16^. These results suggest that assessing PA may indirectly capture elements of systemic inflammation and nutritional imbalance that are not reflected in standard liver function tests.

The graded survival benefit observed across increasing PAI levels suggests a **dose–response relationship** between physical activity and survival. Mechanistically, regular activity may enhance **hepatic perfusion**, modulate systemic inflammation, improve **ammonia clearance**, and stimulate **muscle protein synthesis** via upregulation of anabolic pathways^25–28^. Exercise also reduces portal pressure, improves insulin sensitivity, and attenuates oxidative stress—all mechanisms that may mitigate decompensation risk^27^.

The integration of the RFH-SGA enriches our understanding of the complex interplay between physical activity, nutritional status, and systemic frailty in cirrhosis. This integrated functional-nutritional approach strengthens prognostic frameworks and supports personalized prehabilitation strategies beyond conventional liver severity scores. From a clinical perspective, our **risk-tiered classification** offers practical utility. The striking difference in one-year survival—ranging from 89% in low-risk to 18% in very-high-risk groups—supports the integration of PA assessment into routine evaluation. Patients in the high- and very-high-risk categories may benefit from early **multidisciplinary prehabilitation**, structured exercise interventions, nutritional optimization, and prioritized transplant evaluation. Conversely, low-risk patients can be safely monitored with longer follow-up intervals, optimizing healthcare resources.

Our findings extend the evidence base on exercise and cirrhosis by demonstrating that **self-reported physical activity**, even when measured with a brief instrument like IPAQ-SF, retains predictive accuracy comparable to more objective measures such as six-minute walk distance^23^ or handgrip strength^24^. This highlights the practicality of PAI as a **screening tool** in routine hepatology practice, particularly in settings with limited access to specialized testing.

Prior interventional studies have shown that exercise programs in cirrhotic patients are safe and effective in improving muscle mass and aerobic capacity^25–27^. Our results complement these findings by suggesting that even habitual, non-programmed activity confers survival advantages, underscoring the potential of **lifestyle-based interventions** in early disease stages.

Importantly, the prognostic significance of physical activity persisted even after adjustment for sarcopenia and liver disease severity in multivariable analysis. This suggests that the association between low physical activity and mortality cannot be explained solely by reduced muscle mass or more advanced hepatic dysfunction. Rather, habitual physical activity may reflect a broader construct of physiological reserve encompassing cardiopulmonary fitness, systemic inflammation, metabolic resilience, functional capacity, and frailty. Emerging evidence indicates that frailty and impaired physical performance independently predict hospitalization, decompensation, waitlist mortality, and post-transplant outcomes in cirrhosis, even after accounting for MELD and sarcopenia measures^21,23,28^. Similarly, studies evaluating gait speed, exercise tolerance, and cardiorespiratory fitness have demonstrated prognostic value beyond conventional hepatic severity indices^4,6,23^. These findings support the concept that physical activity captures prognostic dimensions not fully represented by standard liver scores or static assessments of muscle quantity alone. Accordingly, routine assessment of physical activity may provide clinically meaningful complementary prognostic information beyond sarcopenia evaluation in patients with cirrhosis.

The major strengths of this study include its **prospective design**, **objective quantification of sarcopenia** via CT imaging, and **integration of functional and biochemical measures** into a unified prognostic framework. The use of IPAQ-SF ensures accessibility and feasibility in clinical practice.

However, several limitations should be acknowledged. First, PA was assessed through **self-report**, which may introduce recall bias; future studies should incorporate objective measures such as accelerometry. Second, the single-center design and inclusion of patients from a tertiary hepatology referral center may limit extrapolation of findings to community-based cirrhosis populations. Third, **etiological diversity** (e.g., viral, alcoholic, or NAFLD-related cirrhosis) was not stratified in subgroup analyses and could influence PA patterns. Finally, while we identified strong associations, **causal inference** cannot be established in an observational design. Importantly, PAI, CT-derived skeletal muscle index, and liver disease severity scores were assessed at baseline enrollment prior to follow-up initiation. Serial longitudinal reassessment during the 12-month follow-up period was not systematically performed; therefore, the prognostic impact of temporal changes in these variables could not be evaluated.

Future multicenter studies and randomized trials should validate the prognostic role of PAI across broader populations and investigate whether **structured exercise programs** can actively modify outcomes. Integrating wearable-based monitoring and digital health platforms could refine real-time activity tracking and enable personalized prehabilitation strategies before transplantation.

## Conclusion

This study establishes physical activity as a **powerful**,** independent predictor of mortality** in cirrhosis. The IPAQ-SF–derived PAI enhances traditional prognostic models by incorporating a functional dimension reflecting physiological reserve and frailty. Incorporating PA assessment into routine clinical evaluation could refine risk stratification, inform clinical decision-making, and guide the implementation of targeted interventions aimed at improving survival and quality of life.

## Data Availability

“Data is available upon request from the corresponding author.”

## Abbreviations

Cox model: Cox proportional hazards regression
Child–Pugh: Child–turcotte–pugh score
CI: Confidence interval
CT: Computed tomography
HR: Hazard ratio
IPAQ-SF: International physical activity questionnaire–short form
IQR: Interquartile range
LREs: Liver-related events
MELD: Model for end-stage liver disease
PA: Physical activity
PAI: Physical activity index
RFH-SGA: Royal Free Hospital-Subjective Global Assessment
SGA: Subjective Global Assessment
SMI: Skeletal muscle index
SD: Standard deviation
SPSS: Statistical package for the social sciences
